# Work Motivation and Reactions to Injustice of Temporary Workers: Roles of Social Identities, Autonomy, and Compensations

**DOI:** 10.5964/ejop.3755

**Published:** 2022-11-30

**Authors:** Florent Lheureux, Clément Parmentier

**Affiliations:** 1 Laboratoire de Psychologie (UR 3188), Université Bourgogne Franche-Comté, Besançon, France; Central European University, Vienna, Austria

**Keywords:** dual social identification, work motivation, organisational injustice, contingent work, autonomy and compensations

## Abstract

This article addresses the impact of temporary employment on workers’ social identification, work motivation, and reactions to injustice at the workplace. More precisely, we examined whether organisational identification mediates the effect temporary work (compared to permanent employment) on work motivation, and reactions to injustice. We also examined whether autonomy in contract-choice and compensating features of job contracts (employment duration, qualification matching, and negotiated wages) have positive effects on the organisational and ingroup identifications of temporary workers. Finally, we examined whether ingroup identification of temporary workers act as a mediator and moderates the effect of organisational identification. Results from a survey comparing agency workers with fixed-term and permanent employees mainly from the industry sector first reveal that organisational identification mediates the negative effect of temporary work on work motivation and its positive association with self-centred reactions to injustice. Nevertheless, cluster analysis revealed the existence of three subgroups of agency workers, a minority of them—autonomous and compensated—having similarly high levels of identification and motivation than permanent employees. Additionally, autonomous and compensated workers identify more with their ingroup than low-autonomy and low-compensations workers, ingroup identification explaining their difference in terms of work motivation. Furthermore, ingroup identification of agency workers interact with organisational identification to determine their reactions to injustice. Implications, limitations, and research perspectives deriving from this study are discussed.

“Temporary employment” (or “contingent employment”) is an umbrella expression which refers mainly to four types of job contracts: fixed-term, casual, seasonal, and arranged through temporary employment agencies (some other contracts exist depending on countries’ legislations). All can be defined as “dependent employment of limited duration” (De Cuyper et al., 2008, p. 2). Although there were many changes during the last two decades in terms of acceptance of temporary work and more protective regulations (Guest, Isaksson, & De Witte, 2010; Schiek, 2004), temporary workers benefit on average of fewer rights and of lower wages in comparison to permanent employees (Dekker & Wilthagen, 2014; Eurofound, 2015; Pavlopoulos, 2013). There are also differences between temporary workers, with casual and agency workers having less guarantee of employment duration and /or no guarantee of indemnity when dismissed in comparison to fixed-term employees.

Compared to the traditional employment relationship (i.e., full-time and permanent) temporary work can be viewed as more flexible and cost-effective, because it makes organisations abler to adapt their productivity to contingent market fluctuations (e.g., Bosch, 2004). In consequence, it has strongly increased in most industrialized countries over the last four decades (Eurofound, 2015; Golden & Appelbaum, 1992). However, several studies have challenged this view, assuming that temporary work presents “hidden costs” in comparison to permanent employment: lower health status, higher risk of work-related injuries, and reduced levels of attachment to the organisation and effort expended (Allan, 2000; Damiani et al., 2016; Virtanen et al., 2005). Thus, understanding “why” and “when” temporary employment can be associated with negative or positive outcomes is a critical issue that have stimulated abundant studies with many conceptual and theoretical approaches (Cheng, & Chan, 2008; De Cuyper et al., 2008; Wilkin, 2013).

In spite of the vitality of this research domain, the study of the social identity processes associated with temporary work in comparison to permanent work remain in its infancy. In this respect, this article offers new insights on that topic. It examines: 1) whether temporary employment can have a detrimental effect on workers’ identification, work motivation, and reactions to organisational injustice (identification playing a mediating role); 2) whether autonomy in contract-choice and compensating features of job contracts foster organisational identification and; 3) it addresses the role of the dual identification of temporary workers (i.e., with the organisation and with their ingroup of temporary workers).

## Social Identity Processes and the Effects of Temporary Versus Permanent Job Contracts

Since two decades, scarce but insightful studies have investigated the interplay between social identity processes and differences in job contracts (Buonocore, 2010; Chattopadhyay & George, 2001; de Gilder, 2014; George & Chattopadhyay, 2005; Jiang, 2015; Riketta et al., 2006; Rousseau, 1998; Veenstra et al., 2004; von Hippel, 2006). They referred to the Social Identity Approach (SIA, Hogg et al., 2017; Reicher et al., 2010), which considers that individuals are motivated to have a positive, non-ambiguous, and stable personal identity in response to basic self-enhancement and uncertainty reduction needs. One common and effective way to fulfil these needs is to belong to social groups that have a clear, secure, and positive identity in comparison to other groups. Accordingly, studies referring to this approach in occupational /organisational contexts consider that the profession, the organisation, and the work-team individuals belong are major sources of social identities.

For the SIA, social identification is the key process that links together the self and the target social category (e.g., an organisation), the self being defined (self-concept) and valued (self-esteem) in reference to the stereotype of that group (self-stereotyping). When the ingroup is perceived as having a high status in comparison to an outgroup (i.e., a higher prestige, more positive attributes etc.), individuals identify strongly to their group and adopt attitudes and behaviours favouring its members, while derogating outgroup members. This identification to the ingroup is strengthened when the individual feels explicitly valued for his/her contributions to the group and thinks he/she was granted a full /typical membership status (Bartel & Dutton, 2001; Meeussen, & van Dijk, 2016). In contrast, when the ingroup is perceived as having a low status, individuals may be motivated to leave it to enter a more valued outgroup (i.e., a social mobility strategy, Jackson et al., 1996; Tajfel et al., 1979). In this case, identification with the outgroup can be a strong motivator of behaviour (Lheureux & Auzoult, 2017; Munniksma et al., 2015).

Thus, referring to the SIA and in line with the “hidden costs” perspective, Veenstra et al. (2004) and Buonocore (2010) argued that temporary employment hinders these social identification processes. Veenstra et al. (2004) has put forward that temporary workers, (1) cannot anticipate ongoing contact with their colleagues and thus cannot develop a secure sense of inclusion or belonging (low perceived employment security) and, (2) cannot perceive themselves as full /typical members of their work team /organisation (low in-group membership status). In addition, Buonocore (2010)—in line with the theoretical analysis of Rousseau (1998)—considered that organisational identification depends on the “particularistic rewards” the employee received from the organisation (e.g., status, reputation, work arrangements, sharing of important /confidential information). However, because these particularistic rewards are provided to trusted employees and because trust takes time to develop, temporary workers are less likely than permanent employees to receive such type of rewards. Consequently, temporary workers generally identify less with the organisation than permanent employees and, as a result, generally display lower levels of affective commitment and work motivation (i.e., low organisational identification mediates the negative effects of temporary work on these outcomes, Buonocore, 2010; Veenstra et al., 2004). This mediation effect of organisational identification, can be explained by the fact that the higher the social identification is, the more people are inclined to adopt group-serving attitudes and behaviours, because of the tight association of the Self with the target social category (Lee et al., 2015; Reicher et al., 2010). In this respect, permanent employees are likely to have a higher work motivation than temporary workers, because their stronger organisational identification increase their willingness to contribute to the effectiveness of the organisation. Nonetheless, this mediation effect is probably partial, because other mediating mechanisms have been evidenced (e.g., social exchange processes, differences in working conditions, De Cuyper et al., 2008; Hünefeld et al., 2020). Thus, in line with these elements, we put forward the following hypotheses:

- Temporary workers present lower levels of organisational identification (**Hypothesis 1a**) and work motivation (**Hypothesis 1b**) than permanent employees, the negative effect of temporary employment on work motivation (in comparison to permanent employment) being partially mediated by a decrease in organisational identification (**Hypothesis 1c**).

In addition, the Veenstra et al. (2004) study has investigated the impact of temporary work on how employees react to organisational injustice. More precisely, they asked a sample of permanent employees to rate their willingness to adopt four reactions if unfairly treated by a manager, after being experimentally assigned to different scenarios (with a casual, fixed-term or permanent role in a team that had, or did not have, a future). Two reactions were more active /problem-solving oriented (i.e., the *collective response* “getting together with other members of their team and bringing the issue to the attention of another manager” and the *individual response* “raising the issue individually with another manager”). The other two reactions were more passive /problem-avoidance oriented (i.e., the *ignore reaction* “ignoring the problem and getting on with the job” and the *resign reaction* “contemplating resigning from their job”). Moreover, two of these reactions were more self-centred (i.e., trying to solve individually the problem or leaving the organisation), while the other two were more organisation-centred (i.e., trying to collectively solve the problem or compliantly accepting how the organisation function by ignoring it). The implicit rationale of Veenstra et al. (2004) was that because temporary workers identify less with the organisation than permanent employees, they are less willing to invest in a collective response and thus are more likely to react otherwise. However, their results were generally non-significant. The only significant main effect that emerged was that workers assigned to the casual role scenario were more willing to resign than those assigned to a fixed-term or to a permanent role. The pattern of results for the collective response reaction was more complex and did not allow any straightforward conclusion. In our view, this is because they did not survey *actual* temporary workers but permanent employees experimentally assigned to hypothetical scenarios. In fact, we can consider that comparing real temporary workers to permanent employees currently working into the same organisation is more likely to reveal the effect of temporary work on reactions to injustice, as well as the mediating role of organisational identification at this level. More precisely, because high social identification is known to favour collective action and protest, whereas low identification increases an individualistic /self-centred and/or passive approach (Kelloway et al., 2010; Klandermans et al., 2002), we propose the following hypotheses:

- When facing an organisational injustice, temporary workers are less likely to engage in a collective response (**Hypothesis 2a**) and more likely to engage in avoidant and/or self-centred reactions (**Hypothesis 2b**) than permanent employees.
- The negative effect of temporary employment (in comparison to permanent employment) on the collective response and its positive effect on self-centred and /or avoidant reactions are partially mediated by a decrease in organisational identification (**Hypothesis 2c**).

## Examining the Roles of Autonomy, Compensations, and Ingroup Identification of Temporary Workers

In contrast with findings showing that temporary work has a detrimental effect on workers’ organisational identification and related-outcomes, several studies have highlighted that some features of the employing organisation can counterbalance this impact (Buonocore, 2010; George & Chattopadhyay, 2005; Jiang, 2015). More precisely, temporary employment is less likely to have negative effects on identification when workers perceive the organisation as prestigious and distinctive, when they perceive few discriminations from colleagues and supervisors, as well as when they positively perceive the social climate and interpersonal relationships within the organisation.

In the same vein, the study reported below addresses additional factors that may have an impact on temporary workers’ organisational identification. In particular, empirical studies referring to the social identity approach generally ignore the well-established fact that subgroups of temporary workers exist. Therefore, our study aims at improving knowledge at this level, by identifying subgroups of temporary workers for which the detrimental effects of their job contract on organisational identification and its related outcomes would be not observed in comparison to permanent contracts. These factors, as much detailed below, are autonomy in contract-choice and compensations for having a temporary contract.

In fact, the hidden costs perspective has received mixed evidence mainly because temporary workers are far from being a homogeneous group (Gracia et al., 2011). Globally, more positive outcomes were observed for fixed-term, longer duration, qualification-matching, high wage, and voluntarily chosen temporary contracts (i.e., for compensated and autonomous temporary workers), whereas negative outcomes were more prevalent for agency and casual work contracts, chosen under constraint, with shorter duration, low paid, and matching little the qualifications (i.e., for low-compensated /low-autonomy temporary workers) (Busk et al., 2017; Campbell, 2004; Chambel & Castanheira, 2007; Dawson et al., 2017; De Cuyper & De Witte, 2007; Feldman et al., 1995; Krausz, 2000).

Acknowledging this heterogeneity, some studies provided typologies of temporary workers on the basis of their level of autonomy/volition in contract choice and on the presence or absence of compensations for a less secure job (de Jong et al., 2009; Engellandt & Riphahn, 2005; Feldman, 2006; Glaymann, 2010; Marler et al., 2002). When combined together, these typologies allow the differentiation of three sub-types of temporary workers:

- *Involuntary* workers (involuntary with no perceived opportunity for change and with no compensations).
- *Transitory* workers (preferring permanent work but accepting a temporary job with low compensations as a useful short-term option for dealing with a life-change situation, such as a career break, a home move, etc.).
- *Voluntary* workers (choosing temporary work because they consider it as enriching their resume, as well as providing them more freedom and higher wages than permanent employment, because of their distinctive marketable skills and qualifications).

Involuntary and transitory temporary workers represent the majority of this population, while voluntary temporary workers constitute a minority (Glaymann & Grima, 2008; Guest, 2004; Mattijssen & Pavlopoulos, 2019). In line with past research (Bartel & Dutton, 2001; Chattopadhyay et al., 2004; de Gilder, 2014; Riketta et al., 2006) we can consider that compensating features increase the identity-related value of temporary work in the employment marketplace. Longer contract duration can be viewed as a “sign” that social mobility to the desired group (permanent employees) is feasible (intergroup permeability) in exchange of effort and loyalty to the organisation. Higher wages and being hired for a job that match the qualifications can be viewed as particularistic rewards that recognise the specific skills and contributions of the employee to the organisation and as a symbol of membership granting. Likewise, higher wages can be viewed as a source of positive distinctiveness with other employees (i.e., favourable social comparison). Moreover, organisations giving to individuals the possibility to choose an employment contract that match their preferences (i.e., autonomy in contract-choice) are more likely to generate positive feelings and judgments (e.g., Chambel & Castanheira, 2007; Krausz, 2000). Conversely, we can consider that in absence of autonomy and compensations, nothing can counterpoise the inherent low job security and peripheral membership status associated with temporary employment in comparison to permanent employment (as stated by Veenstra et al., 2004).

Following this rationale, we postulate that:

- Temporary workers having low-autonomy and low-compensations (i.e., involuntary and transitory) present lower levels of organisational identification (**Hypothesis 3a**) and work motivation (**Hypothesis 3b**) than permanent employees, whereas voluntary temporary workers (i.e., autonomous and compensated) are similar to permanent employees concerning these outcomes.
- The decrease in organisational identification mediates the strength of their differences with permanent employees in terms of work motivation, such that the lower identification of low-autonomy /low-compensated workers in comparison to voluntary workers explain their stronger differences with permanent employees (**Hypothesis 3c**).

This study also addresses another theoretical issue concerning the social identity processes of temporary workers: The neglected role of their ingroup identification. As research on dual identification suggests (e.g., George & Chattopadhyay, 2005; Sluss & Ashforth, 2008; Vora & Kostova, 2007), the client organisation is not the unique focus of identification for temporary workers. For instance, they can also identify with the temporary work agency, the two foci of identification (i.e., the agency and the client organisation) being potentially salient at the same time, associated and might interact to determine outcomes (George & Chattopadhyay, 2005). However, in spite of its potential effects on attitudinal and behavioural outcomes, past research have not considered the role of the ingroup identification of temporary workers, in addition to other foci of identification. Accordingly, our study aims at providing preliminary information regarding its determinants and consequences, alongside organisational identification.

The aforementioned rationale suggests that autonomy and compensations can also have a positive impact on temporary workers’ ingroup identification. In cases of low-autonomy and low-compensations, temporary workers are likely to have a low ingroup identification because they perceived their group as having a lower status than permanent employees, this situation generating outgroup favouritism and “social mobility” strategies toward the group of permanent employees (von Hippel, 2006). Conversely, temporary jobs with compensating features and matching the preferences of workers are likely to increase the perceived status of temporary workers. Thus, in this case, the more they perceive their ingroup as a source of positive identity—alternative to the organisation—the less they are likely to adopt an organisation-centred approach to injustice (i.e., less trying to collectively solve the problem or compliantly accepting how the organisation function), while the more they probably adopt an avoidant self-centred approach (i.e., leaving the organisation). In other words, in cases of organisational injustice, temporary workers’ ingroup identification is likely to have effects opposite to that of organisational identification.

Besides, temporary workers’ ingroup identification probably moderates the effects of organisational identification on their reactions to injustice. For workers that strongly identify with the organisation, injustice is likely to be experienced as a major threat to personal identity, because of the strong association of the Self with the unjust organisation (Kelloway et al., 2010). In this case, the degree with which temporary workers valued their ingroup and identify with it probably act as a buffer against threat, because in this case their ingroup offers a positive substitute identity. This probably facilitates their psychological detachment from the organisation in cases of injustice. Accordingly, dual identifiers (i.e., those having jointly high levels of ingroup and organisational identifications) are more likely to leave the unjust organisation (i.e., to resign), because for them it is not perceived as the sole available basis for building a positive identity. Conversely, if temporary workers are dependent of the organisation to satisfy their needs of belonging and of collective self-esteem (i.e., if they do not value their ingroup of temporary workers), they are less likely to resign because of a lack of a positive substitute identity. In addition, for them, the only way to protect and to restore their social identity is to engage in a collective response to injustice (a perspective consistent with the Identity Threat Theory of Petriglieri, 2011; see also Holmes IV et al., 2016). By contrast, dual identifiers are less likely to collectively respond to injustice, because this strategy is not their unique possibility to preserve their positive identity. This view is consistent with research illustrating the advantages of having multiple identities: it makes people “able to switch among different social identities according to one’s current context, needs, or goals” (Kang & Bodenhausen, 2015, p.561). This is for instance the case of studies showing that identity switching efficiently helps to deal with stereotype threat, as identifying oneself to an alternative ingroup not targeted by the negative stereotype eliminate the threat to personal identity (Rydell, McConnell, & Beilock, 2009).

All these elements converge to delineate the following hypotheses:

- Autonomous and compensated temporary workers (i.e., voluntary) identify more with their ingroup than those low in autonomy and compensations (transitory and involuntary) (**Hypothesis 4a**).
- The more temporary workers identify with their ingroup the less they adopt problem-solving organisation-centred reactions to injustice (e.g., a collective response), whereas the more they adopt avoidant self-centred reactions (e.g., resigning) (**Hypothesis 4b**).
- The increase in ingroup identification mediates the differences of voluntary workers with low-autonomy /low-compensated workers in terms of collective response (negative indirect effect) and of avoidant /or self-centred reactions (resign, ignore, individual response) (positive indirect effect) (**Hypothesis 4c**).
- The higher the temporary workers’ ingroup identification is, the less organisational identification favours a collective response and the more it favours a resign reaction (**Hypothesis 4d**).

**Figure 1:**
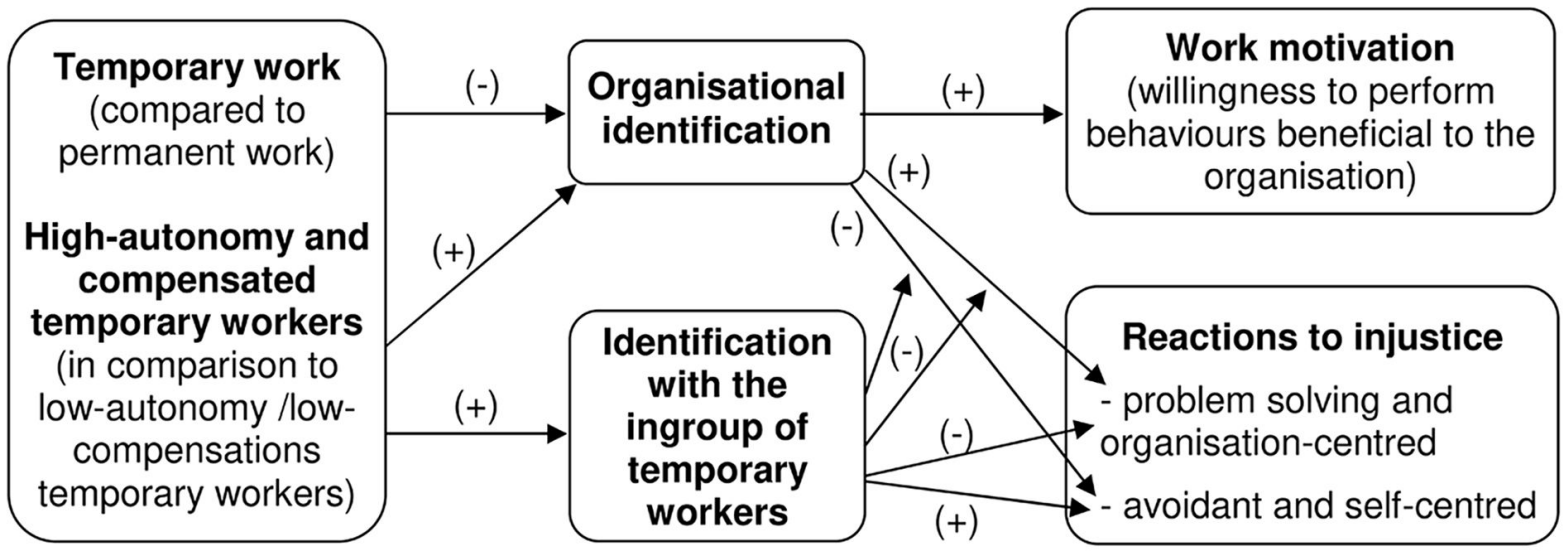
Graphical Representation of the Hypotheses Under Study *Note*. (+) is for “positive effect” and (-) is for “negative effect”.

All the above-mentioned hypotheses are summarized in Figure 1. In order to empirically test these ones, we conducted a survey comparing samples of actual agency workers, fixed-term, and permanent employees with regards to the target variables. We have especially investigated the heterogeneity of agency workers in terms of autonomy in contract-choice and in terms of the presence/absence of compensations, in order to assess whether these factors have an impact on their organisational and ingroup identifications and, consequently, on work motivation and reactions to injustice.

## Method

### Participants and Procedure

We include samples of agency workers, fixed-term and permanent employees. Because agency work is by far the least secure type of job contract in France (Glaymann, 2010), we had a specific focus on the autonomy-related motives and compensating features of job contracts of agency workers. Because we expected to compare fixed-term and permanent employees with up to three subgroups of agency workers, power analysis for a one-way ANOVA with five groups was conducted to determine a sufficient sample size using an alpha of 0.05, a power of 0.80, and a medium effect size (*f*^2^ = 0.25) (following the procedure of Faul et al., 2009). According to these assumptions, the required minimum sample size was 200.

Participants were recruited via two procedures. First, several directors of temporary work agencies of the district of our university were contacted. They allowed us to distribute a questionnaire to the workers coming to the agency over a one-and-a-half-month period. They were invited to complete it at home and to deposit it in a locker reserved for this purpose during their next visit. In addition, to ensure that agency workers would be compared to permanent and fixed-term employees that worked in the same client organisations, managers from firms that employ most of our agency worker participants allowed us to distribute the survey, using the same completion and retrieval procedure. In all, 617 questionnaires were distributed and 232 (37.60%) were completed. Three participants were excluded due to too many missing values (> 10%).

As a result, a total of 229 participants, mainly from the industry sector (*n* = 201) and secondarily from the construction, retailing, and socio-cultural activity sectors constitute the final sample. Sixty-nine were permanent employees, 52 were fixed-term employees and 108 were agency workers. No differences between the three groups were observed with regards to occupational and demographic variables, excepting for age, for permanent employees *M* = 33.87; for fixed-term employees *M* = 26.85; for agency workers *M* = 27.91, *F*(2) = 15.49, *p* < .0001, (for more details regarding the sample composition see the Supplementary Materials).

### Material

#### Measures Common to All Participants

All participants first completed the items below. These items were rated on a 7-point agree-disagree Likert scale.

##### Work Motivation

We used 6 items from Veenstra et al. (2004), which cover typical /traditional measures (i.e., commitment, effort, unpaid overtime) and non-typical measures (i.e., error identification, creativity, compliance to managers’ instructions) to broaden the range of behaviours that are susceptible to be performed by highly motivated employees. Only five items were combined to obtain a reliable overall measure of work motivation (α = .72), given that the “creativity” item did not load with other items on the expected “work motivation” factor in a principal component analysis (see Supplementary Materials section).

##### Organisational Identification

We used six items (α = .90), including three items about the work team and three items that relate to the company. We used the four items of Veenstra et al. (2004) plus two other items to increase the number of items per identification foci (i.e., when I talk about my current work group, I use “us” rather than the “them”; when I talk about my current company, I use “us” rather than the “them”).

##### Reactions to Organisational Injustice

In line with Veenstra et al. (2004), participants indicated their willingness to adopt four types of reactions if treated unfairly by a manager: (a) getting together with other members of their team and bringing the issue to the attention of another manager (*collective response*, which is a problem-solving organisation-centred reaction); (b) raising the issue individually with another manager (*individual response*, which is a problem-solving self-centred reaction); (c) trying to ignore the problem and get on with the job (*ignore reaction*, which is an avoidant organisation-centred reaction) and; (d) contemplating resigning from their job (*resign reaction*, which is an avoidant self-centred reaction).

#### Measures Specific to Agency Workers

To account for the heterogeneity of agency workers in terms of autonomy in contract-choice and compensating features, as well as to investigate their differences in terms of ingroup identification, they replied to the additional specific measures below.

##### Autonomy-Related Motives for Being Agency Workers

Agency workers also had to choose from a list of motives for being agency workers, those which best described their situation (max. 4 answers). These items were inductively derived from the sociological work of Glaymann (2010) who has investigated the diversity of French agency workers’ autonomy-related motives. Three measured a constrained choice /involuntary situation (e.g., I didn’t choose agency work; α = .77), three other items measured a transitory /contingent choice (e.g., I have chosen agency work because I’m pending for another job or for a home move; α = .81), and the remaining items measured a voluntary choice (e.g., I have chosen agency work because I like change and to be independent; α = .67). This scale obtained for each motives type a score ranging from 0 (no selected item) to 3 (all selected).

##### Compensating Features and Ingroup Identification

Finally, they also rated their average employment duration (1 = *one week or less*; 2 = *between 1 week and 1 month*; 3 = *between 1 month and 6 months*; 4 = *more than 6 months*), the average frequency with which their employment matched their qualifications (1 = *rarely*; 2 = *often*; 3 = *always*), the frequency of wages negotiation when hired (0 = *never*; 1 = *infrequently*; 2 = *sometimes*; 3 = *frequently*) and their identification with the agency workers group (using items similar to those used to measure organisational identification, α = .86).

### Data Analyses

More details regarding data analyses are available in the Supplementary Materials. We first performed a principal component analysis with promax rotation to ensure that the organisational identification and work motivation scales constituted homogeneous and distinct—though correlated—measures. Then, we examined the descriptive statistics and bivariate correlations between variables. Hence, we used a hierarchical ascending cluster analysis to differentiate agency workers on the basis of their autonomy-related motives for being agency worker and on the presence or absence of compensating features (contract duration, qualification matching and salary negotiation) (Murtagh & Contreras, 2012). We next used ANOVAs to compare the different groups of participants regarding social identification and outcome variables. Finally, to examine the different mediation and moderation hypotheses we used conditional process analyses (Hayes, 2017). At this level, we not only consider the exclusion of zero to interpret the presence of an indirect effect, but we also consider the Relative Proximity (RP) index, which is “the distance to zero of an indirect effect’s CI as a function of its range” (Götz et al., 2021, p.97). This standardised statistic allows for direct comparisons of several CI regarding their proximity or distance to zero: the higher the value, the stronger the indirect effect is across bootstrapped samples, whereas a positive value close to zero (< .10 in this article) suggests a very low and/or very unstable indirect effect. In addition, we estimate the observed statistical power for each reported indirect effect, using the procedure of Schoemann et al. (2017), with a value of at least .80 considered as satisfactory.

## Results

**Figure 2:**
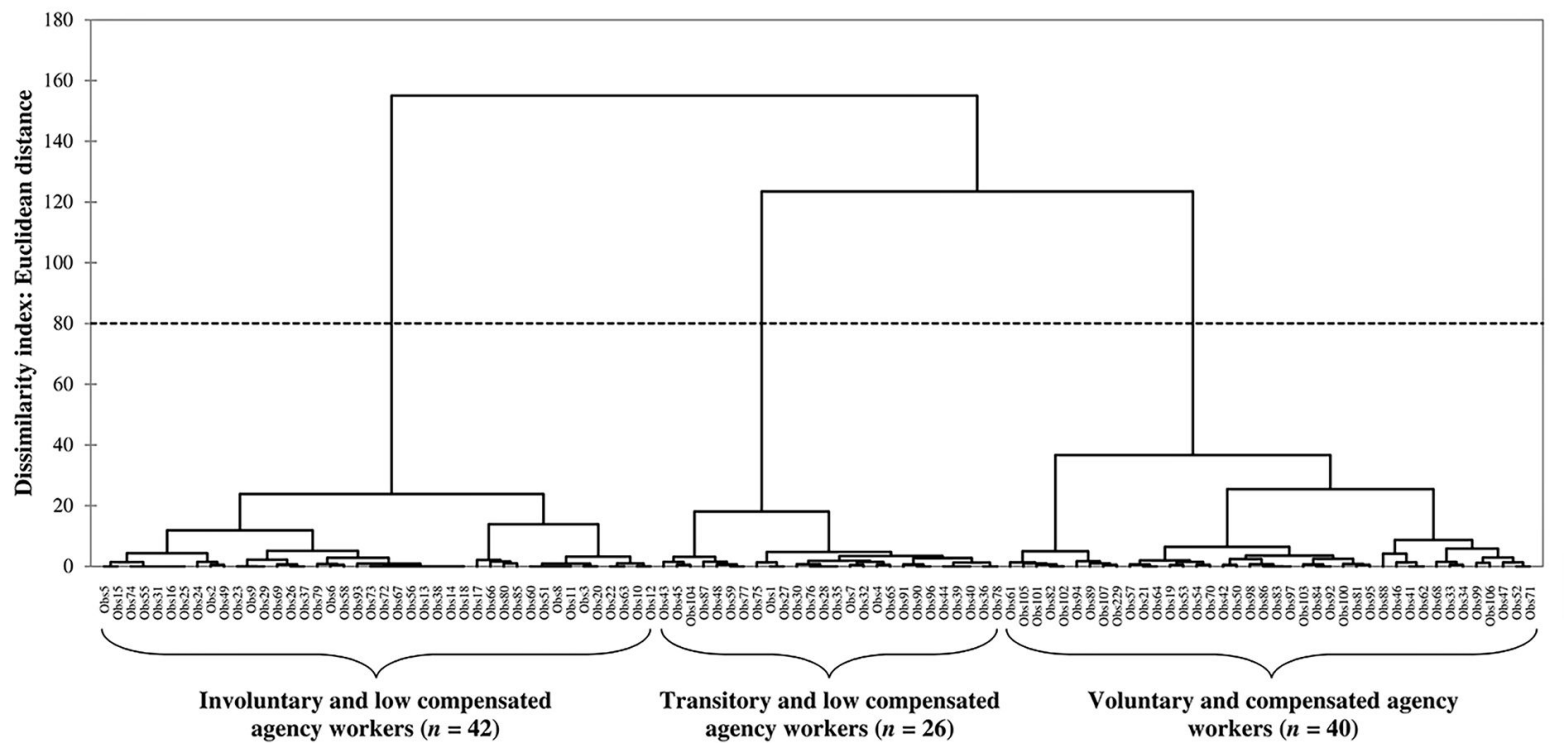
Dendrogram From a Hierarchical Ascending Cluster Analysis of Agency Workers on the Basis of Their Autonomy-Related Motives and on the Presence/Absence of Compensating Features (Ward’s Aggregation Method)

Table 1 displays descriptive statistics and main bivariate correlations between variables. Consistent with the typology described in the introduction, the cluster analysis revealed three main clusters of agency workers (see Figure 2). The larger cluster is that of involuntary agency workers (*n* = 42): they reported low levels of employment duration, qualification matching, and wages negotiation and felt constrained, agency work being for them a default-option. The next group is that of transitory agency workers (*n* = 26): they also reported low levels of employment duration, qualification matching, and wages negotiation but accept agency work to accommodate a life-change period. Together, these two groups constitute the majority of agency workers (*n* = 69, 64%) and can be considered as low-compensated /low-autonomy. Finally, voluntary agency workers (*n* = 40) reported higher levels of compensations and had chosen agency work mainly because they consider it as a lifestyle with its own advantages (for details see the Supplementary Materials).

Concerning ANOVAs (Table 2), when agency workers are considered together (Model 1) they had *on average* lower levels of organisational identification (*M* = 4.00) and work motivation (*M* = 4.16) than permanent (*M* = 5.53 and *M* = 4.88 respectively) and fixed-term employees (*M* = 5.30 and *M* = 4.75 respectively) (*p* < .05 for all comparisons according to Tamhane’s T2 test; all omnibus effects were significant, Welch’s corrected *F*(2) ≥ 16.99, *p* ≤ .0001, with adjusted *R^2^* values ranging from .13 to .43). This is consistent with the “hidden costs” perspective of the effects of temporary employment (H1a and H1b).

However, these mean differences were largely due to the fact that the majority of agency workers (about 64%) reported low levels of autonomy and compensations. In fact, when the three types of agency workers were distinguished (Model 2) the *R*^2^ values significantly increased for four variables in comparison to Model 1 (i.e., Δ*R^2^* ranging from .04 to .27; all omnibus effects were significant, with Welch’s corrected *F*(4) ≥ 17.43, *p* < .0001). Consistent with hypotheses 3a and 3b, voluntary workers obtained higher scores (*p* < .05) than low-autonomy/low-compensated workers (i.e., transitory and involuntary) for organisational identification (*M* = 4.68, *M* = 3.64 and *M* = 3.56 respectively) and for work motivation (*M* = 5.00, *M* = 3.98 and *M* = 3.47 respectively). Thus, as expected, autonomy and compensations globally reduced the differences of agency workers with fixed-term and permanent employees for these variables. However, concerning organisational identification, the differences of permanent (*M* = 5.53, *p* < .0001) and fixed-term employees (*M* = 5.30, *p* < .001) with voluntary workers remained significant.

**Table 1:** Descriptive Statistics, Reliability Coefficients When Relevant (Cronbach’s Alpha in Parentheses), and Main Bivariate Correlations

N°	Variable	*N*	*MV*	*M* or %	*SD*	9	10	11	12	13	14	15	16	17	18
1	Age	229	0	29.46	8.44	.11	.15*	.11	.02	-.28**	.00	-.16	.18	.19**	.23*
2	Gender ^a^ (women)	228	1	22%	0.42	-.07	-.09	.02	.03	.05	.13	-.03	.04	-.06	-.13
3	Position ^b^ (manager)	198	21	1.19	0.42	.08	.01	.17**	-.06	-.06	.04	.04	-.12	.03	-.21*
4	Education level	206	23	3.83	0.67	.17*	.04	.01	.04	-.07	-.24*	.17	.04	.11	.08
5	Full-time job? ^c^	229	0	93%	0.26	.06	.03	.27**	-.12	-.01	-.08	-.05	.02	-.00	-.15
6	Average employment duration	108	0	2.55	0.77	.39**	.18	-.21*	.14	-.38**	-.43**	.09	.23*	.39**	.33**
7	Average frequency of qualification matching	108	0	1.94	0.89	.42**	.04	.02	-.16	-.15	-.09	-.19	.17	.38**	.28**
8	Average frequency of salary negotiation	108	0	0.32	0.58	.39**	.03	-.20*	.38**	-.26**	-.35**	-.11	.50**	.52**	.54**
9	Work motivation	229	0	4.51	0.89	(.72)	.08	.15*	-.23**	-.23**	-.43**	-.09	.27**	.59**	.46**
10	Individual response	228	1	4.31	1.48			-.01	-.05	-.30**	-.22*	.40**	-.20*	.10	.21*
11	Collective response	227	2	3.91	1.20				-.32**	-.13*	.16	-.11	-.02	.29**	-.30**
12	Resign reaction	226	3	2.08	1.55					.05	.03	-.13	.37**	-.25**	.40**
13	Ignore injustice reaction	229	0	4.84	1.38						.32**	-.18	-.25**	-.26**	-.31**
14	Involuntary motives	108	0	1.06	1.08						(.71)	-.46**	-.33**	-.23*	-.27**
15	Transitory motives	108	0	0.80	1.19							(.88)	-.21*	-.17	-.18
16	Voluntary motives	108	0	0.55	0.80								(.67)	.32**	.44**
17	Organisational identification	226	3	4.76	1.10									(.90)	.57**
18	Agency workers ingroup identification	108	0	4.18	1.33										(.86)

*Note.* MV = missing values; ^a^ Male = 1, Female = 2; ^b^ Subordinate = 1, manager = 2; ^c^ No = 1, Yes = 2.

*p < .05. **p < .01.

Concerning reactions to organisational injustice, a repeated measures ANOVA showed a significant interaction between the reaction type variable (within-subject) and the type of job contract variable (between-subject), *F*(5.72) = 42.07, *p* < .0001 (using the Greenhouse-Geisser correction because of a violation of the sphericity assumption; Mauchly’s test = 0.93, *p* = .004).

More precisely—and partially consistent with H2a—we observed that permanent employees were more likely to collectively respond to injustice (*M* = 4.72) than fixed-term (*M* = 4.12, *p* = .007) and agency workers (*M* = 3.29, *p* < .0001), but were also strongly inclined to respond individually (*M* = 4.99). In line with the Sub-Hypothesis H2b, agency workers considered together and fixed-term employees were more willing to resign (*M* = 3.01 and *M* = 1.48 respectively) and to ignore injustice (*M* = 5.04 and *M* = 5.58 respectively) than permanent employees (*M* = 1.06 and 3.99 respectively, all *p* < .0001).

Conditional process analyses provided results globally consistent with mediation and moderation hypotheses, with some noteworthy exceptions (more details are available on request). First, consistent with the Hypothesis 1c, the negative association of agency work with work motivation was significantly mediated by a reduced organisational identification, *b* = -0.75, *SE* = 0.13, 95% CI [-1.01, -0.52], RP = 1.06, 1−β = 1.00. A significant but lower negative mediated effect of the fixed-term job contract was also observed, *b* = -0.11, *SE* = 0.05, 95% CI [-0.21, -0.01], 1−β = .99, but a low RP value of 0.05. When the three subgroups of agency workers having different levels of autonomy and compensations were differentiated (Hypothesis 3c), mediated effects of organisational identification on work motivation were significant for all these groups, but were of greater magnitude for involuntary workers, *b* = -0.61, *SE* = 0.14, 95% CI [-0.92, -0.35], RP = 0.61, 1−β = 1.00, and transitory workers, *b* = -0.59, *SE* = 0.14, 95% CI [-0.87, -0.33], RP = 0.83, 1−β = .1.00, than for voluntary workers, *b* = -0.26, *SE* = 0.07, 95% CI [-0.41, -0.14], RP = 0.52, but a very low power 1−β of .08. Thus, the greater decrease in organisational identification for low-autonomy and low-compensations workers than for voluntary workers explained their greater differences with permanent employees in terms work motivation.

**Table 2:** Results (Estimated Means, Standard Deviations, Adjusted R^2^ Values, and Post-hoc Tests) From Analyses of Variance Comparing Permanent Employees and Fixed-Term Employees With all Agency Workers Considered Together (Model 1) or With the Three Types of Agency Workers Identified in Cluster Analysis (Model 2)

	Estimated Mean (*SD*)								
	Agency Workers				Other Employees		adj. *R*^2^		
Variable	All AWs	Involuntary low comp.	Transitory low comp.	Voluntary compensated	Fixed-term	Permanent	Model 1	Model 2	Δ adj.*R^2^*
Organisational Identification	^b^4.00 (1.05)	3.56_c_ (1.09)	3.64_c_ (0.85)	4.68_b_ (0.74)	^a^ 5.30^#^_a_ (0.63)	^a^ 5.53^#^_a_ (0.49)	.43	.54	.11
Work Motivation	^b^ 4.16 (1.07)	3.47 _b_ (0.91)	3.98 _b_ (0.59)	5.00^#^_a_ (0.91)	^a^ 4.75^#^_a_ (0.35)	^a^ 4.88^#^_a_ (0.62)	.13	.40	.27
Ignore Injustice	^b^ 5.04 (1.21)	5.57^#^_a_ (1.15)	4.73 _b_ 0.83)	4.68 _bc_ (1.29)	^b^ 5.58^#^_a_ (0.78)	^c^ 3.99 _c_ (1.57)	.18	.22	.04
Resign Reaction	^a^ 3.01^#^ (1.79)	3.15^#^_a_ (1.40)	2.50^#^_a_ (1.42)	3.20^#^_a_ (2.27)	^b^ 1.48 _b_ (0.54)	^c^ 1.06 _c_ 0.24)	.33	.34	.01
Individual Response	^b^ 4.29 (1.41)	3.81 _b_ (1.33)	5.27^#^_a_ (0.92)	4.15 _b_ (1.46)	^c^ 3.46 _b_ (1.24)	^a^ 4.99^#^_a_ (1.42)	.13	.20	.07
Collective Response	^c^ 3.29 (1.06)	3.48 _bcd_ (1.17)	3.00 _cd_ (1.06)	3.28 _cd_ (0.91)	^b^ 4.12 _b_ (1.20)	^a^ 4.72^#^_a_ (0.83)	.26	.27	.01
Agency Workers Ingroup Identification	4.18 (1.33)	3.64 _b_ (1.33)	3.74 _b_ (0.97)	5.03^#^_a_ (1.33)	**/**	**/**	/	.23	/

*Note*. AW = Agency Worker Means sharing a common superscript (Model 1) or subscript (Model 2) were not statistically different at α = .05 according to Tamhane’s T2 test. Highest values are marked with a superscript hash sign (with “a” superscripts or subscripts). Δ*R^2^* = Model 2 *R^2^* - Model 1 *R^2^*. All Welch’s corrected *F* values (not reported here) and all Δadj.*R^2^* values ≥ .04 were significant at *p* < .05.

Partially contradicting Hypothesis 2c, there were no significant indirect effects for the individual and collective responses to injustice. For the two avoidant reactions indirect effects were significant, but it was positive for the ignore reaction, agency workers: *b* = 0.48, *SE* = 0.17, 95% CI [0.17, 0.83], RP = 0.26, 1−β = .95; fixed-term employees: *b* = 0.07, *SE* = 0.04, 95% CI [0.01, 0.17], 1−β = .99, but a low RP value of 0.06, and negative for the resign reaction, agency workers: *b* = -0.52, *SE* = 0.21, 95% CI [-0.91, -0.10], RP = 0.12, 1−β = .89; fixed-term employees: *b* = -0.08, *SE* = 0.05, 95% CI [-0.19, -0.002], 1−β = .87, but a low RP value of 0.01. Thus, these results revealed that for temporary workers (especially agency workers) organisational identification motivates a higher tendency to ignore the injustice and a lower tendency to resign in comparison to permanent employees. Interestingly, for the resign reaction the direct effect was opposite (i.e., positive), when controlling for the negative indirect effect. This suggests that another variable intervene in explaining the difference between temporary workers and permanent employees at this level. This variable is probably the ingroup identification of temporary workers.

In fact, as expected by the Hypothesis 4a, voluntary workers (i.e., autonomous and compensated) have a significantly higher ingroup identification (*M* = 5.03), than transitory workers (*M* = 3.84, *p* < .0001) and involuntary workers (*M* = 3.74, *p* < .0001) (see Table 3). Additionally, in line with Hypothesis 4b, ingroup identification was positively associated with self-centred reactions to injustice, resign: *b* = 0.75, *SE* = 0.16, 95% CI [0.43, 1.06]; individual response: *b* = 0.31, *SE* = 0.13, 95% CI [0.06, 0.56], while negatively associated with the collective response, *b* = -0.38, *SE* = 0.10, 95% CI [-0.58, -0.19]. However, it was not significantly associated with the ignore reaction, *b* = -0.13, *SE* = 0.11, 95% CI [-0.36, 0.08]. Moreover, in line with Hypothesis 4c, ingroup identification significantly mediated the positive association of the voluntary workers’ group with the individual response, *b* = 0.43, *SE* = 0.19, 95% CI [0.03, 0.82], but a low RP value of 0.04 and a low power 1−β of .63, and the resign reaction, *b* = 1.04, *SE* = 0.29, 95% CI [0.54, 1.69], RP = 1.15, 1−β = .95, as well as its negative association with the collective response, *b* = -0.53, *SE* = 0.15, 95% CI [-0.82, -0.24], RP = 0.41, 1−β = .89. Finally, as expected by Hypothesis 4d, there were two significant interaction effects of organisational identification and ingroup identification, on the resign reaction, *b* = 0.27, *SE* = 0.11, 95% CI [0.05, 0.48], RP = 0.12, and on the collective response, *b* = -0.15, *SE* = 0.07, 95% CI [-0.29, -0.01], but a low RP value of 0.04.

**Table 3:** Status of Each Hypothesis According to Empirical Results

Hypotheses		Status
H1a	Negative effect of temporary work on organisational identification.	Partially corroborated (for agency workers)
H1b	Negative effect of temporary work on work motivation.	Partially corroborated (for agency workers)
H1c	The decrease in organisational identification partially mediates the negative effect of temporary work on work motivation.	Corroborated (but low RP for fixed-term employees)
H2a	Negative effect of temporary work on the collective response to injustice.	Corroborated
H2b	Positive effect of temporary work on avoidant and/or self-centred reactions to injustice.	Partially corroborated (for avoidant reactions)
H2c	The decrease in organisational identification partially mediates the effect of temporary work on reactions to injustice (negative effect on the collective response, positive effect on avoidant and /or self-centred reactions).	Partially corroborated (for avoidant reactions, but negative effect for resigning and low RP values for fixed-term)
H3a	Low-autonomy and low-compensations temporary workers identify less to the organisation than permanent employees, while voluntary temporary workers identify similarly to them.	Partially corroborated (reduced but still sig. differences with permanent employees for voluntary workers)
H3b	Low-autonomy and low-compensations temporary workers have a lower work motivation than permanent employees, while voluntary temporary workers are similarly motivated.	Corroborated
H3c	The decrease in organisational identification mediates the strength of their differences with permanent employees in terms of work motivation, the lower identification of low-autonomy /low-compensated workers than voluntary workers explaining their higher differences with permanent employees.	Corroborated (but very low statistical power for voluntary workers)
H4a	Voluntary temporary workers identify more with their ingroup than those low in autonomy and compensations.	Corroborated
H4b	Negative effect of ingroup identification of temporary workers on problem-solving organisation-centred reactions to injustice (collective response) and positive effects on avoidant /or self-centred reactions (resign, ignore, individual response).	Partially corroborated (non-significant for ignore)
H4c	The higher voluntary workers’ ingroup identification mediates their differences with low-autonomy /low-compensated workers in terms of collective response (negative indirect effect) and avoidant or self-centred reactions (positive effects).	Corroborated (but low statistical power for the individual response)
H4d	The higher the temporary workers’ ingroup identification is, the less organisational identification favours a collective response and the more its favours a resign reaction.	Corroborated (but low RP for the collective response)

As illustrated by Figures 3a and 3b, a high identification with the ingroup of agency workers completely reversed the effect of organisational identification (i.e., decreasing the collective response and increasing the resign reaction). Thus, dual identifiers were less willing to collectively solve the problem and more willing to leave the organisation, while agency workers that identified with the client organisation only were more willing to engage in collective action and less willing to resign.

**Figure 3a:**
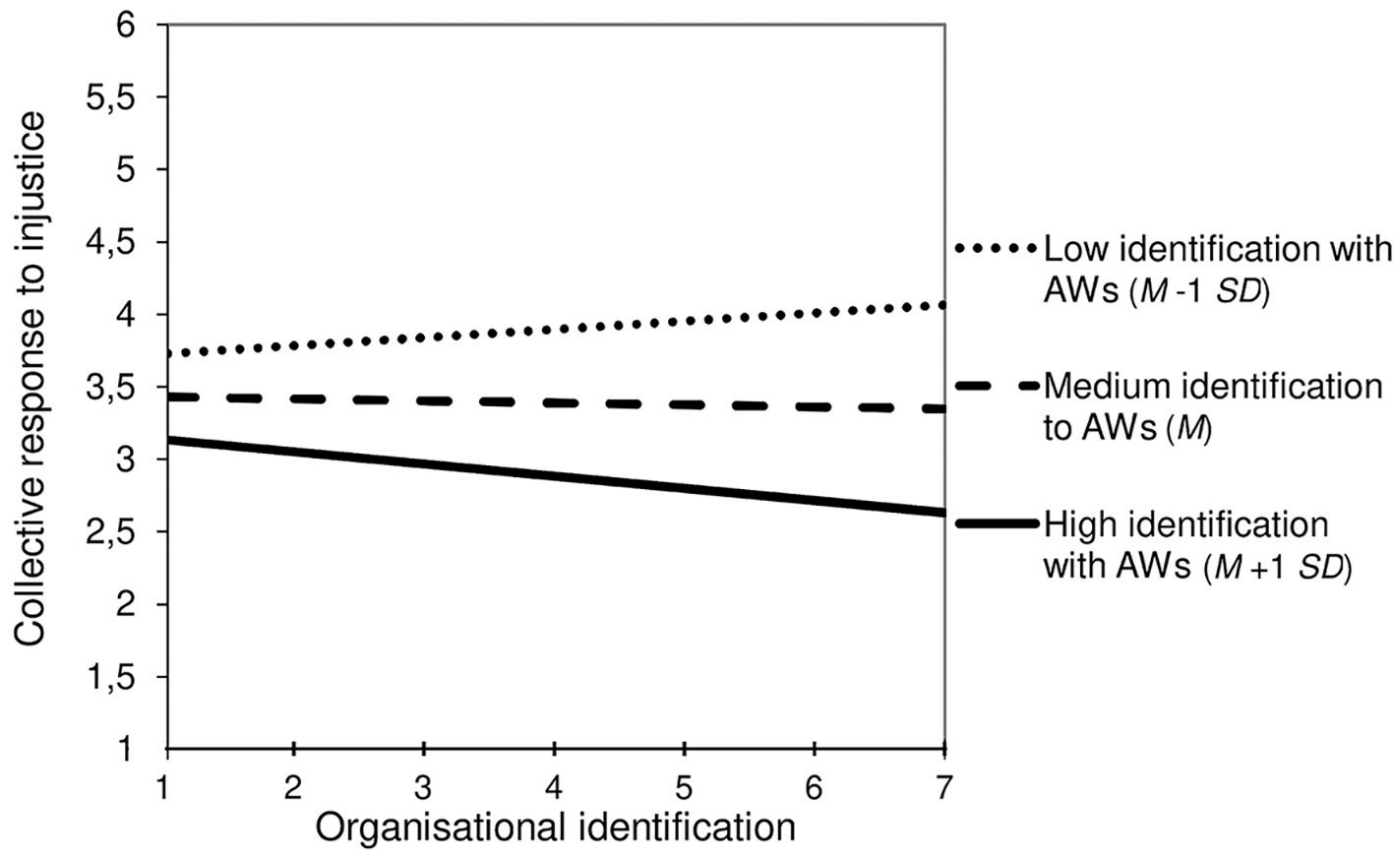
Agency Workers’ (AWs) Ingroup Identification Moderates the Relationships of Organisational Identification With the Collective Response to Organisational Injustice (Simple Slopes)

**Figure 3b:**
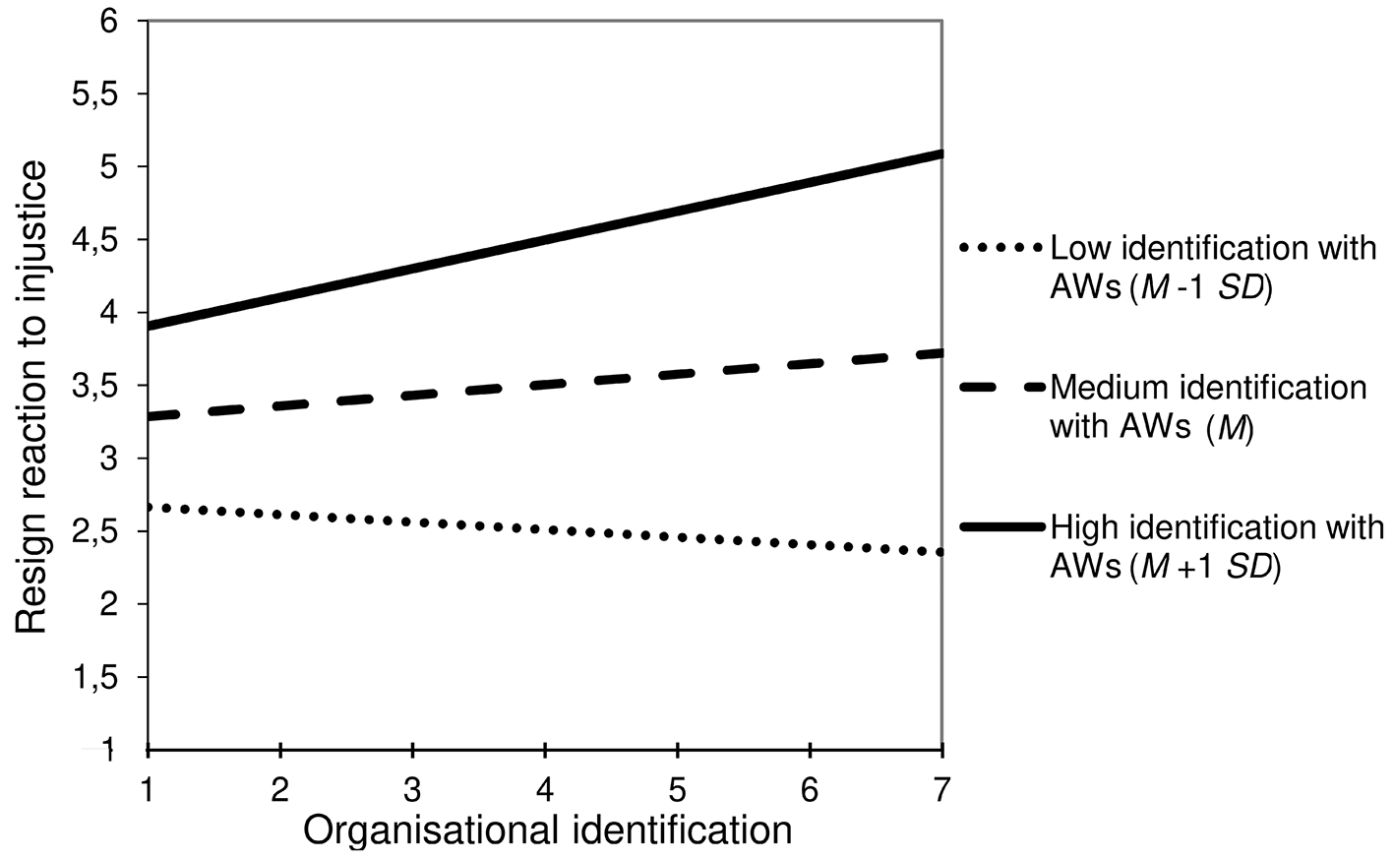
Agency Workers’ (AWs) Ingroup Identification Moderates the Relationships of Organisational Identification With the Resign Reaction to Organisational Injustice (Simple Slopes)

## Discussion

### Summary of Findings and Study Contributions to Knowledge

Most hypotheses received full or partial support from the results (see Table 3), although with some statistical limitations for few of them (low RP or power values, see the “limitations” subsection). First, in line with the hidden cost perspective (e.g., Veenstra et al., 2004), the results illustrated that agency work is *on average* associated with lower organisational identification and lower work motivation than permanent work (as expected by Hypotheses 1a and 1b) (Table 2). However, agency workers with autonomy and compensations (i.e., voluntary) had a higher organisational identification and a higher work motivation than low-autonomy and low-compensations workers (i.e., involuntary and transitory), in line with Hypotheses 3a and 3b. Thus, for a minority of agency workers (autonomous and compensated) the hidden costs are reduced, as their differences with permanent employees were lowered. Thus, in addition to features of the employing organisation (i.e., high prestige, low perceived discrimination, positive social climate, Buonocore, 2010; George & Chattopadhyay, 2005; Jiang, 2015), factors that differentiate subgroups of workers also have an impact on organisational identification. Additionally, the positive association of organisational identification with work motivation was consistent with past research (Meyer et al., 2006; Riketta, 2005).

These results are consistent with the idea that compensating features can be viewed as particularistic rewards and symbols of membership granting (Bartel & Dutton, 2001; Buonocore, 2010) that value the social identity of temporary workers. The significant positive correlations between compensating features and agency workers’ ingroup identification corroborate this perspective (Table 1). Besides, as expected by Hypothesis 4a, we observed that voluntary workers strongly identified with the agency workers’ group because this type of job contracts matched more their career preferences (high autonomy in contract choice) and offered them more advantageous compensations (e.g., higher wages) than permanent employment, whereas other subgroups of agency workers identified less with their ingroup because they perceived it as having a lower status than permanent employees (von Hippel, 2006) (Table 2).

This study also contributes to research on how employees react to organisational injustice (Table 3, Figures 3a, and 3b). First, it is the first study that illustrates the mediating role of organisational identification regarding the impact of temporary employment on avoidant reactions to workplace injustice (i.e., ignore and resign reactions). The more they identify with the organisation the less they resign (in line with H2c), but the more they ignore injustice (contrary to H2c); perhaps because this injustice was not described as directed to the participant and perhaps because organisational identification may maintain a trusting and loyal attitude to the organisation (Al-Shalabi, 2019). Furthermore, permanent employment fosters problem-solving reactions more than temporary work, especially the collective response (in line with H2a), but this effect was not mediated by organisational identification (which contradicts H2c).

At this level, our results revealed for the first time the role of the ingroup identification of temporary workers (expected by H4b, H4c, and H4d): The more they identify with their ingroup the more they adopt self-centred reactions and the less they collectively respond to injustice, ingroup identification mediating the effect of temporary work on these reactions. Furthermore, ingroup identification mediated the association of the voluntary workers’ subgroup with a more self-centred and avoidant approach to injustice (i.e., resign). In addition, agency workers’ ingroup identification moderated the effect of organisational identification on the collective response and resign reaction (as expected by H4d, see Figures 3a, and 3b). Accordingly, organisational identification increased the willingness to collectively respond to injustice and decreased the willingness to leave the organisation, excepting when agency workers strongly identified with their ingroup. In this case, the opposite pattern was observed: Agency workers that strongly identified with the client organisation were more willing to resign and less willing to engage in a collective action. Thus, our results suggest the existence of a “double-edged sword effect” of the dual identification of agency workers: on the one hand they've got the same motivation at work than permanent employees, but on the other hand they are strongly inclined to leave the organisation if they feel treated unfairly. This is probably because organisational injustice is more likely to be experienced as a threat to personal identity when workers identify strongly with the organisation (Kelloway et al., 2010). In this case, the choice between the “fight” (collective response) or the “flight” (resign) reactions depends on the degree with which agency workers perceive their ingroup as an alternative source of positive identity (a result consistent with Identity Threat Theory, Holmes IV et al., 2016; Petriglieri, 2011).

### Study Limitations and Conclusion

Despite its merits, this study suffers from some limitations. First of all, inferences regarding causal relationships between variables were limited because of the use of a cross-sectional design. Nevertheless, this design was suited to examine for the first time the roles of ingroup identification, autonomy, and compensations regarding the effect of temporary work on organisational identification, work motivation, and reactions to injustice. Additionally, the sample was relatively small and mainly came from the industry sector. These elements limit the generalizability of our results, which require to be replicated on larger and more diverse samples. However, these results are relatively free of confounding effects, as the sampling procedure allowed us to compare agency workers, fixed-term, and permanent employees from the same organisations. Additionally, as summarized by Table 3, some results require to be examined in larger sample to increase statistical power. This would also help to determine whether the low RP values observed for some results were really due to this sample size (which may have limited the precise estimation of low magnitude indirect effects) or if any ignored important moderator variable must be identified to explain the range of CI. Anyway, until such studies are conducted, these results must be cautiously considered as relevant but pending further evidence. Finally, although this has favoured the comparability of our study with Veenstra et al. (2004), the use of their work motivation scale has limited the integration of our results in the mainstream research on work motivation. Thus, any subsequent research should use another work motivation scale (e.g., that of Wright, 2004) to ensure the robustness of our findings across different measures.

Keeping these limitations in mind, this study contributed to knowledge in several ways concerning, 1) the impact of temporary work on work motivation and reactions to organisational injustice (in comparison to permanent employment), 2) the differentiation of subtypes of temporary workers according to their perceived autonomy and compensations for a less secure job contract and, 3) the role of social identification processes in this context. As a result, it suggests several avenues for future research and important practical implications can derive from it (see Supplementary Materials for more details).

## Supplementary Materials

For this article the following Supplementary Materials are available via PsychArchives (for access see the Index of Supplementary Materials below)

- Additional statistical tables for the various studies, definitions and explanation of study concepts and results

LheureuxF.
ParmentierC.
 (2022). Supplementary materials to "Work motivation and reactions to injustice of temporary workers: Roles of social identities, autonomy, and compensations"
[Additional definitions, statistical tables]. PsychOpen. 10.23668/psycharchives.12171
PMC978073836605090

## Data Availability

Data is freely available at Supplementary Materials
